# Tuberculosis of the elbow joint: the complexity of diagnosis and treatment—A case report and review of literature

**DOI:** 10.1186/s13256-025-05102-8

**Published:** 2025-03-03

**Authors:** J. Manske, E. Tille, A. Schlüßler, A. Biewener, J. Nowotny

**Affiliations:** University Center for Orthopaedics, Trauma and Plastic Surgery, Shoulder and Elbow Section, University Hospital Carl Gustav Carus, Fetscherstraße 74, 01307 Dresden, Germany

**Keywords:** Elbow tuberculosis, Multidrug-resistant tuberculosis, *Mycobacterium tuberculosis*, Elbow joint, Arthritis, Case report

## Abstract

**Background:**

Tuberculosis is one of the deadliest diseases worldwide, with an estimated incidence of more than 10 million new cases annually. As part of bone and joint tuberculosis (5–6% of all extrapulmonary tuberculosis cases), elbow tuberculosis is a rare manifestation—especially in the Western world—and is associated with nonspecific symptoms such as swelling, redness, and painful limitation of motion. This often leads to initial misdiagnoses, such as septic arthritis or rheumatoid arthritis, resulting in a significant delay in diagnosis and treatment.

**Case presentation:**

A 27-year-old male patient from Bangalore, South India presented with left elbow pain and restricted motion. The clinical and imaging findings led to the suspicion of olecranon bursitis. Intraoperatively, joint tuberculosis was suspected; therefore, multiple tissue samples were taken and a diagnostic routine according to guidelines was initiated. The tuberculosis-specific interferon gamma test was positive and thus confirmed the patient’s previous contact with *Mycobacterium tuberculosis*. Since extrapulmonary tuberculosis is often caused by multidrug-resistant mycobacterial strains, tuberculostatic therapy was started after obtaining the resistogram. Under the initiated therapy, a reduction in synovial inflammation on magnetic resonance imaging and a rehabilitation of the mobility of the elbow were achieved over a period of more than 15 months.

**Conclusion:**

The basis for finding the diagnosis is a detailed, interdisciplinary diagnostic process, especially in patients with persisting unspecific symptoms, since joint tuberculosis is frequently the only site of manifestation. Despite the slow growth of mycobacteria, the microbiological findings, particularly the resistogram, should be awaited since extrapulmonary tuberculosis is often multidrug resistant. As shown in this case, surgical treatment is important for reliable diagnosis, including pathogen identification, but it is not mandatory for successful healing and regaining functionality of the affected joint.

## Background

Tuberculosis (TB) is one of the deadliest diseases worldwide, with an incidence of more than 10 million new cases estimated annually [1]. In recent years, there has been an increase in extrapulmonary tuberculosis outside of known risk areas, mainly owing to migration and traveling, which has led to rising case numbers overall in Europe as well as in Germany [1, 2]. Elbow TB is a rare manifestation of TB, especially in the Western world. It is associated with nonspecific inflammatory symptoms such as swelling, redness, and painful limitation of motion. The unspecific clinical presentation is the reason why there is often a significant delay in the diagnostic process and, accordingly, in the initiation of a specific treatment. This is despite the fact that bone and joint tuberculosis is frequently the only site of manifestation, accounting for 5–6% of all extrapulmonary TB (15–20%) [3]. The key to successful treatment lies in the earliest possible diagnosis, which can be achieved through a detailed anamnesis, an observant breakdown of medical history, extended imaging methods, and interdisciplinary collaboration.

Despite the slow growth of mycobacteria, the resistogram should be awaited since extrapulmonary TB is often caused by multidrug-resistant mycobacteria stems [4–6]. Therefore, long-term antibiotic treatment using individually compiled drug combinations is obligatory. As shown in the underlying case, surgical treatment is not mandatory for successful healing and rehabilitation of joint functionality and should therefore be evaluated critically after weighing the available therapeutic options [7–9].

## Case presentation

**Fig. 1:**
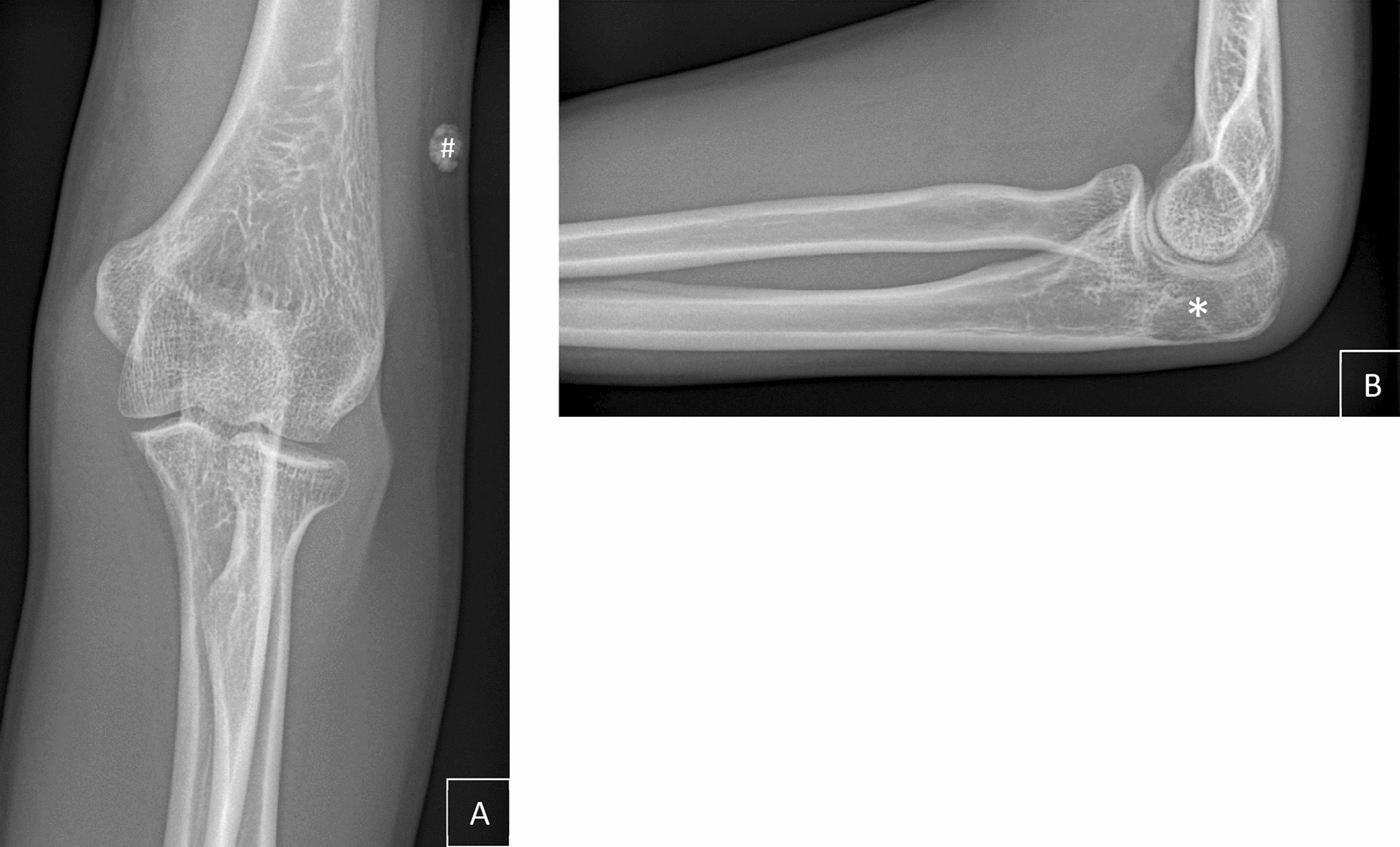
X-ray of the left elbow, taken on 17 April 2020; **A** anterior–posterior view, # shows unclear soft tissue calcification; **B** lateral view, *shows potential osteolysis

A 27-year-old healthy Indian male patient from Bangalore presented in our surgical emergency department in the beginning of June 2020 with a painful limitation of motion in the left elbow. The patient described a fall during which he impacted his elbow, approximately 2 months prior to the initial presentation. No other traumas had occurred recently. An outpatient X-ray diagnosis carried out in April showed no fracture (Fig. 1). The patient stated no other medical conditions, no operations, and no need for long-term medication.

### Clinical findings

The initial clinical examination in June 2020 revealed nonirritant and closed skin conditions and discrete swelling and tenderness over the left olecranon. According to the neutral zero method, there was limitation of motion as follows: flexion/extension of 130°/30°/0° and pronation/supination of 70°/0°/70°.

Aside from the abovementioned findings, there were no other noticeable problems with the left upper extremity. A further body check revealed no findings. Laboratory results showed normal values, except for a slightly elevated C-reactive protein (CrP) of 10.3 mg/L and an elevated total creatine kinase activity (4.86 µmol/s L). Within the externally conducted X-ray of the elbow in two planes, a potential osteolysis of the olecranon was suspected (Fig. 1).

**Fig. 2:**
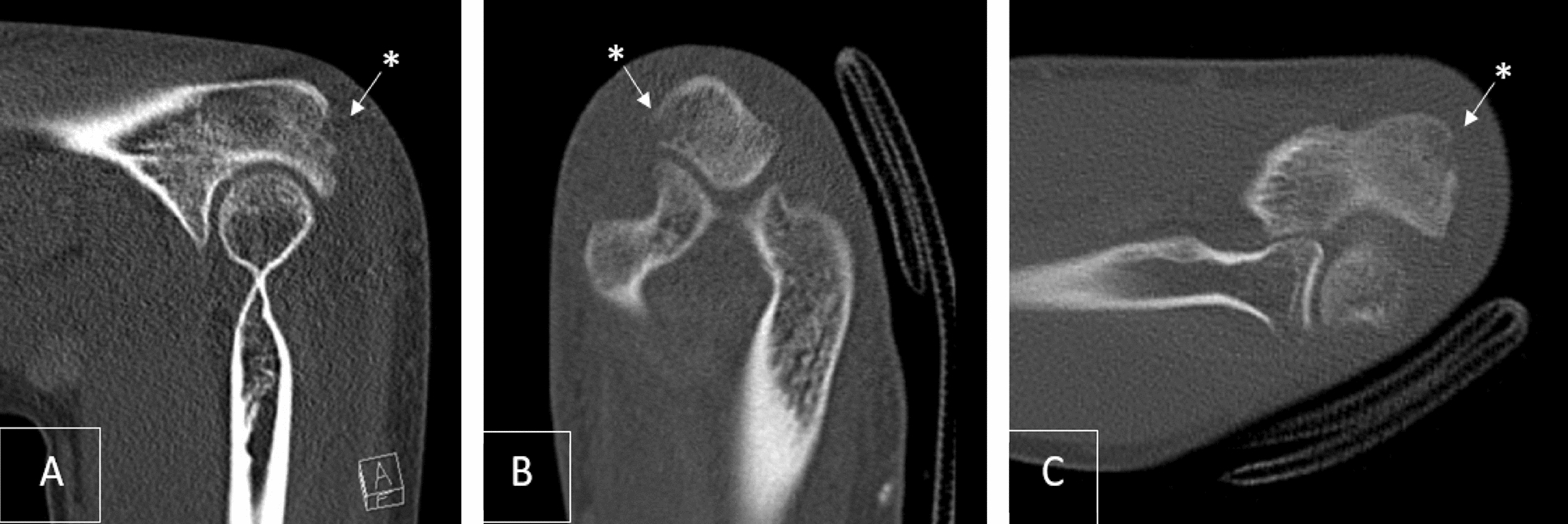
Computed tomography scan of the left olecranon; **A** sagittal; **B** axial; **C** coronal; *shows suspected erosion of the olecranon

Therefore, further imaging with a computed tomography (CT) scan was initiated. The CT revealed an erosion of the proximal olecranon at the insertion of the triceps muscle (Fig. 2), with no confirmation of the suspected osteolysis. The patient was discharged home from the emergency room (ER). The recommendations included early functional exercise without weight-bearing for 3 weeks and regular clinical follow-ups. Beyond that, the use of nonsteroidal antiinflammatory drugs (NSAIDs) (ibuprofen 600 mg up to three times a day) as antiinflammatory and pain therapy was recommended.

**Fig. 3:**
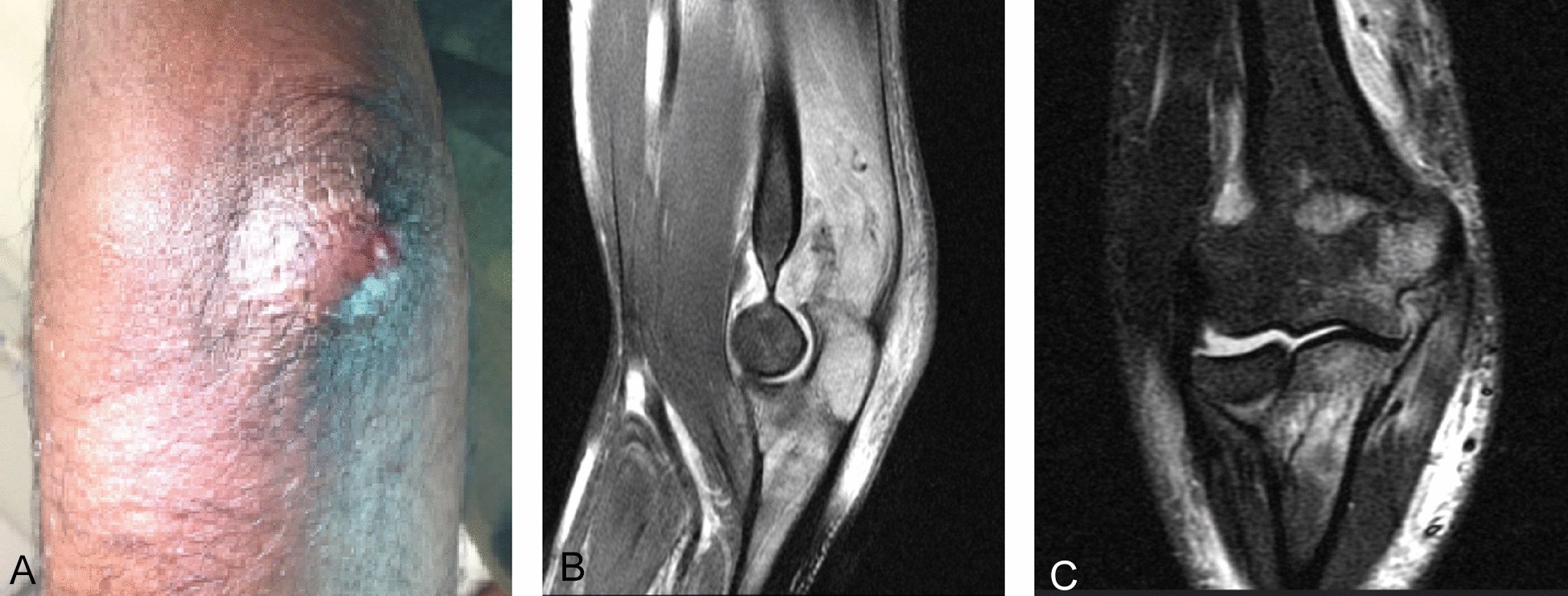
**A** Clinical impression of the olecranon (dorsal, left) on 25 June 2020; **B** and **C** magnetic resonance imaging scan (B, sagittal; C, coronal) with peripheral edema of the proximal olecranon as a sign of a beginning osseous infection

Then, 3 weeks later the patient returned to the hospital for follow-up. This time a perforated purulent bursitis olecrani of the left elbow was suspected (Fig. 3A). The external magnetic resonance imaging (MRI) scan showed the known erosion with significant peripheral edema of the proximal olecranon as a sign of a beginning osseous infection. In addition, a clear joint effusion was visible (Fig. 3B,C). Osteolysis could not be ruled out. Clinically, there were no signs of sepsis (for example, fever and chills). The laboratory findings showed a slightly elevated CrP (12.3 mg/L) accompanied by normal leukocytes (7.1 GPt/L). Owing to reasonable clinical suspicion of burisitis, the patient was admitted to the hospital for surgical treatment, which included a bursectomy and multiple sampling.

Intraoperatively, a 1 cm-sized elliptic skin defect, a 1 cm × 1.5 cm-sized defect of the joint capsule, and a purulent white exudate from the joint were seen. A total of four native, nonformalin-fixed samples were collected for microbial (three) and histopathological (one) assessment. On the basis of these findings, and considering the patient’s origin, joint TB was clinically suspected for the first time. Postoperatively, a standard calculated antibiotic therapy with amoxicillin 2 g/sulbactam 1 g, administered three times per day, was started while microbial and histopathological results were not yet available.

Subsequently, the diagnostic algorithm for TB (chest X-ray, repeated microbial sputum samples, and interferon gamma test), as recommended by the German S2k guideline on the treatment of tuberculosis in adults, was performed [10, 11]. From the abovementioned tests, only the tuberculosis-specific interferon gamma test was positive. Of the three tissue samples collected intraoperatively, only one was found to be populated with *staphylococcus* epidermidis (1/3). In the first sample collection, no mycobacteria were detected. Following the recommendation of our colleagues from the department of infectiology, we did not start tuberculostatic therapy owing to missing results from the histopathological and microbiological examinations. The following days were uneventful. The inserted drainage was removed when the exudate regressed. The inflammatory parameters normalized. The wound was always free of irritation. Further symptoms were not reported by the patient. Then, 5 days later, a second-look surgery was performed with further microbial-specimen collection. The antibiotic therapy was adapted to clindamycin 600 mg three times per day, according to the available resistogram of the detected *staphylococcus* epidermidis.

When the wound was dry and without irritation, the patient was discharged 2 days after the second-look surgery, with immobilization. Wearing the cast until completed wound healing, with daily exercises to maintain mobility, was advised. The antibiotic therapy was recommended to be continued for an additional 5 days. Furthermore, we scheduled regular appointments with our infectious disease outpatient clinic. The results of the histopathological and microbiological examinations were still pending at this time.

**Fig. 4:**
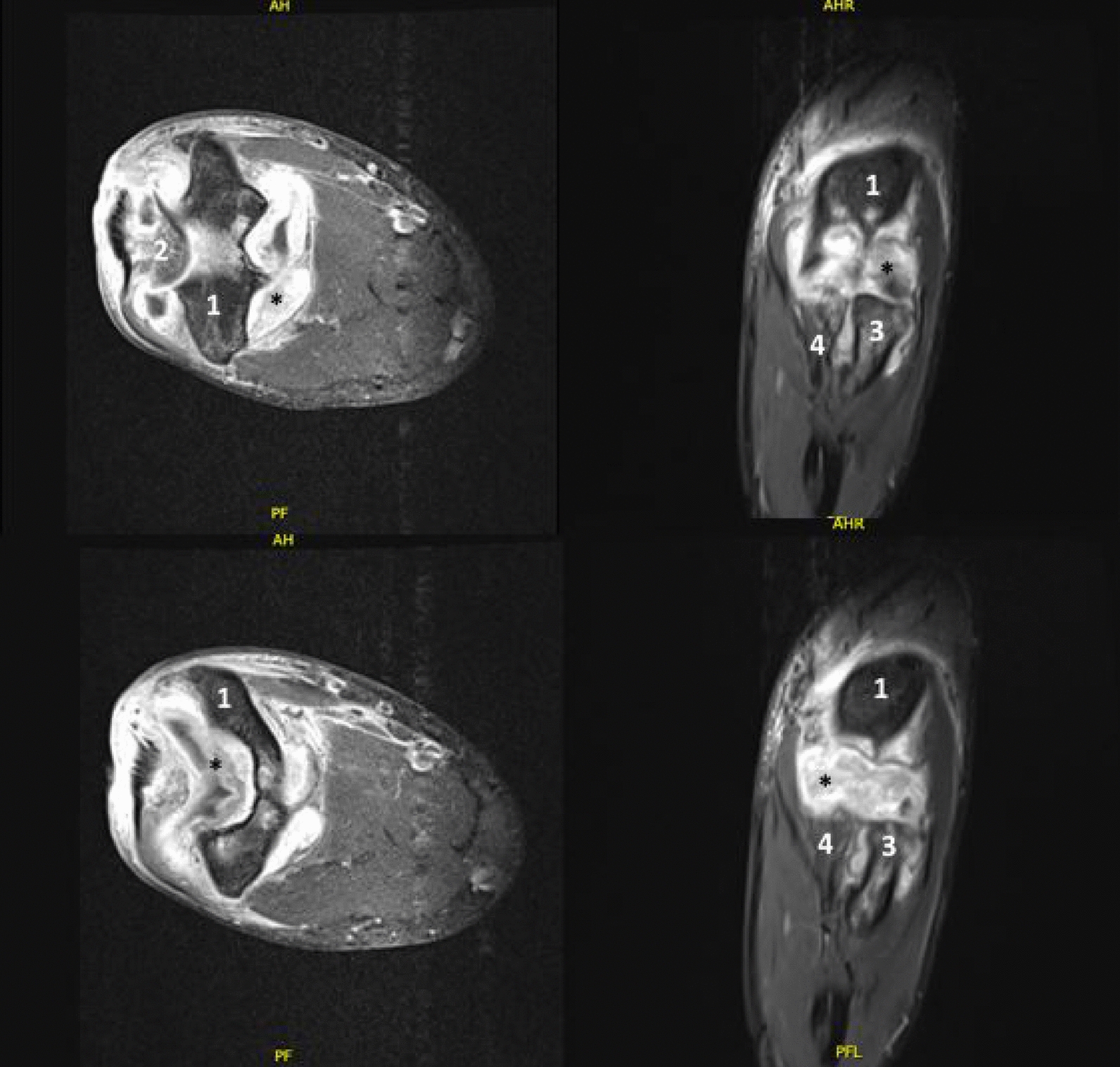
Magnetic resonance imaging scan of the elbow 21 weeks after initial clinical examination: 1, humerus; 2, olecranon; 3, radius; 4, ulnae; *shows synovial inflammation

**Fig. 5:**
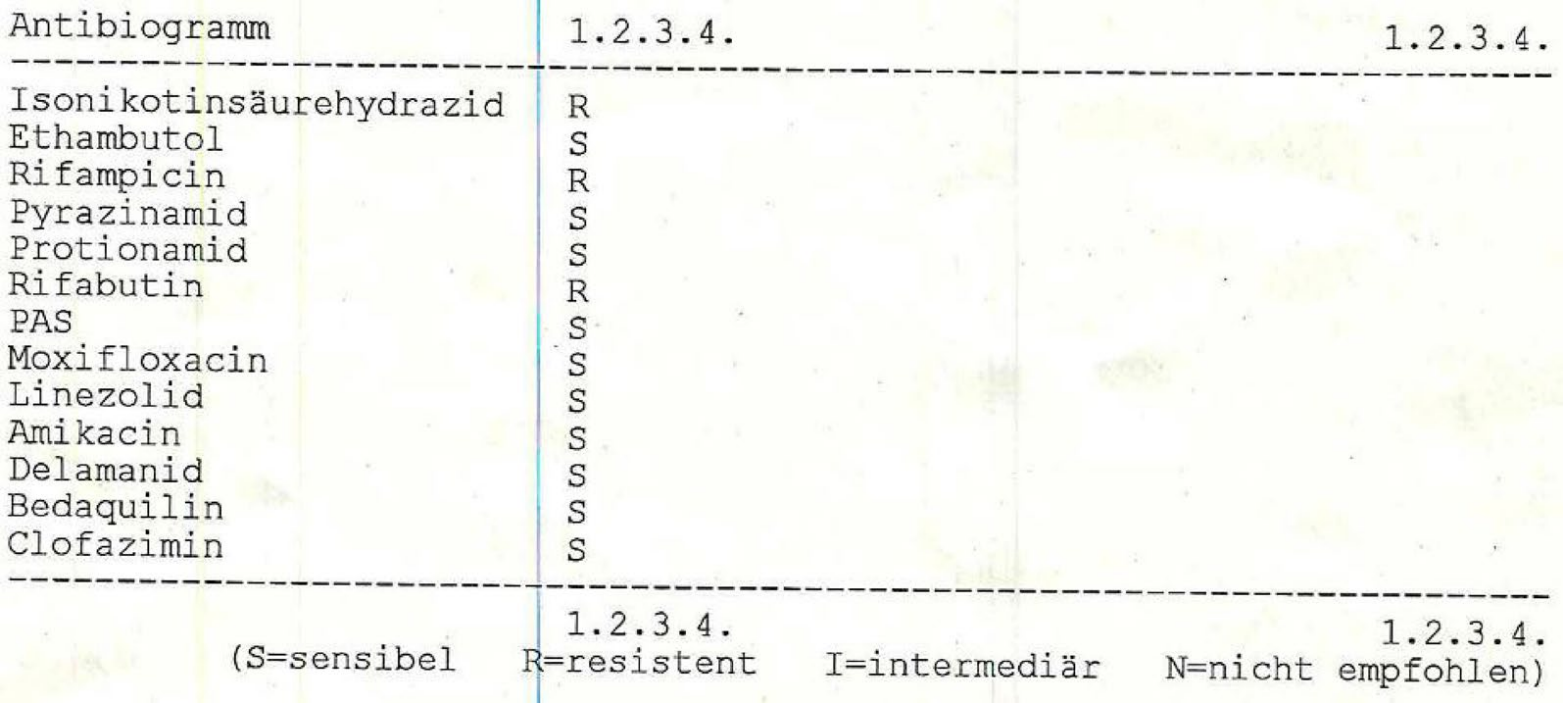
Resistogram from the external laboratory presenting drug resistance against isonicotinic acid-hydrazide, rifampicin, and rifabutin; *R* resistant; *S* sensitive

The verdict of elbow joint TB was first confirmed histopathologically (the species-specific polymerase chain reaction (PCR) (IS6110) for the detection of *M. tuberculosis*, *M. bovis*, and *M. bovis* Bacillus Calmette–Guérin (BCG) proved positive [123-bp amplification]) in the second sample collected during the second-look surgery. Then, 6 weeks after the culture incubation of the tissue samples taken intraoperatively, microbiological evidence of *M. tuberculosis* was obtained. Owing to the still-pending resistogram, there was no change in therapy. In the following weeks, the patient was not available for medical therapy several times owing to personal reasons, hence delaying the clinical follow-up. Finally, 3 months after the operative treatment, the patient presented for another clinical examination on his own initiative. At this point, we found a recurrence of the swelling of the elbow and a new extension deficit of 30°. The scar was dry and nonirritated and the laboratory values displayed no abnormalities. Owing to the extraordinarily slow bacterial growth in the isolate, the resistogram was not available for a long time. Therefore, another follow-up was scheduled. However, 2 weeks later, the clinical findings were still unchanged. Moreover, 4 months after the initial presentation, an increasing effusion in the elbow joint became apparent, so a follow-up MRI was conducted. The MRI (Fig. 4) showed a progressive and extensive synovial inflammation of the entire elbow joint with a rather small joint effusion. Owing to the extremely slow growth of the bacteria and the extensive resistance testing, we did not receive the final resistogram (Fig. 5) until 4 months after initial presentation.

Thereafter, an interdisciplinary case discussion took place. The idea of another surgical treatment to reduce the bacterial load was depraved. In consideration of the finally available resistogram and a joint effusion that was not very pronounced on imaging, it was decided to opt for a solely drug-based therapy. Prior to antibiotic treatment, we conducted routine diagnostic testing, as recommended by the German S2k guideline on treatment of tuberculosis in adults [10]. This included a chest X-ray to detect specific pulmonary changes (already done), an electrocardiogram (ECG), as well as an ophthalmologic consultation. Table 1 gives an overview of possible findings.

**Table 1:** Routine diagnostic testing prior and during antibiotic treatment and possible findings [10]

Routine diagnostic testing	Possible findings
Chest X-ray	Pathological changes in the pulmonary skeleton such as: •nonspecific hilar lymph node enlargement •homogeneous consolidations •unilateral pleural effusions •cavernous tip foci •calcified scars
ECG	QTc time prolongation prior to planned therapy with levofloxacin, bedaquiline, and clofazimine
Ophthalmologic control	Optic neuritis during therapy with ethambutol
Laboratory control	•blood cell count •kidney function parameters (pyrazinamid) •liver function parameters (Isoniazid)

Subsequently, 5 months after the initial presentation, we began the tuberculostatic therapy (bedaquiline, levofloxacin, linezolid, clofazimine, ethambutol, and pyrazinamide) in accordance with the resistogram under frequent laboratory testing of electrolyte levels and renal and hepatic parameters.

The patient was closely monitored with repeated follow-up examinations (laboratory, ECG, ophthalmologic, and imaging controls). During the MRI control, 25 weeks after starting the antibiotical therapy, a regression of the synovialitis was recorded. The tuberculostatic therapy was well tolerated by the patient. With resolution of the synovial inflammatory reaction, the clinical findings also improved. The patient regained a nearly unimpaired range of motion with flexion/extension of 130°/5°/0° and pronation/supination of 90°/0°/90° (Table 2). The swelling and tenderness on palpation over the left elbow resolved during the treatment.

**Table 2:** Overview of the scores and ranges of motion in the course of therapy

Score	Initial clinical examination	5 months after initial clinical examination	8 months after initial clinical examination	32 months after initial clinical examination
OES	–	34	41	43
MEPS	–	65	100	100
DASH	–	31.7	15.0	3.3
Pain on VAS	–	6/10	3–4/10	0/10
Flex./ext.	130°/30°/0°	100°/30°/0°	120°/15°/0°	130°/0°/0°
Pro./sup.	70°/0°/70°	90°/0°/90°	90°/0°/90°	90°/0°/90°

*OES* Oxford Elbow Score, *MEPS* Mayo Elbow Performance Score, *DASH* Disabilities of Arm, Shoulder, and Hand Questionnaire, *VAS* visual analog pain scale, *flex./ext.* flexion and extension according to the neutral zero method, *pro./sup.* pronation and supination according to the neutral zero method

After a slightly longer than 7 months of therapy, the antibiotic regimen was changed to bedaquiline, levofloxacin, ethambutol, and pyrazinamide.

From the beginning of June 2021, the patient experienced adverse drug reactions in the form of numbness and paresthesia (burning sensation) in the region of both forefeet and the soles of the feet. Accordingly, the therapy with ethambutol was paused and switched to clofazimine. With this therapy, the numbness and burning sensations did resolve. After a change of residence to the Netherlands owing to personal reasons, further care was provided there.

Until the end of the therapy in May 2022, numbness of the feet, joint pain, and dry skin remained as side effects. Even after discontinuation of the medication, the patient complained of persistent numbness of the feet and very dry skin. Beyond that, there were no complaints in everyday life. Mobility of the elbow was unrestricted and only occasional pain occurred during heavy exertion.

## Discussion

TB continues to be one of the most common and deadly diseases worldwide, with an overall increasing incidence [1]. In Germany, the incidence per 100,000 inhabitants rose from 5.3 (2010) to 7.1 (2015). The incidence has fallen continuously in recent years, although there is currently (2024) a slight increase again. In 2021, the year in which our case originated, the incidence was 4.7/100,000 inhabitants [12, 13]. Most patients originated from Romania (6.9%), while India was the second most common country of birth (6%) in 2021 [12].

The most common site of manifestation of infection with *M. tuberculosis* is the lung. In only 35% of cases does TB occur extrapulmonary. In these cases, usually lymph nodes, parenchymatic organs, and the spine are affected. Infections of other bones or peripheral joints, such as the elbow, joint are very rare, with an incidence of approximately 2–5% [4, 12, 14–16]. Although the incidence in risk areas, such as the country of origin of our patient, is high at 15–20%, it is comparatively low in the Western world [3, 17]. This is why current literature mainly consists of case reports and a few reviews. Randomized studies evaluating the efficacy of a specific treatment or giving guidance regarding the diagnostic process are scarce, if not unavailable [7].

The aim of this case report is, therefore, to raise awareness of TB affecting peripheral joints, as well as to give a clinical recommendation in synopsis with other international reports. TB of the elbow often presents with nonspecific symptoms of a painful joint, sometimes with swelling and hyperthermia, accompanied by limited range of motion [5, 15, 16].

A systemic reaction such as fever, fatigue, and weight loss occurs very rarely [5]. In many cases, the symptoms initially mimic other diseases such as rheumatic or common septic arthritis [18–20]. Owing to this, many reports mention a significant delay (several weeks to years) until the correct diagnosis is made [5, 7, 21, 22]. In our case, purulent bursitis was the initial verdict. Other case reports describe similar problems. Tangadulrat *et al*. initially diagnosed septic arthritis [7]. Jung *et al*. described a lateral epicondylitis that did not improve with local gating and intramuscular stimulation. Over time, elbow TB was diagnosed [22]. Seung *et al*. described a case in which monoarthritis affecting the hands was mistakenly treated as seronegative rheumatoid arthritis and the correct diagnosis was only made after the therapy did not show any betterment after 1 year [19]. Delayed diagnosis, therefore, not only prolongs the patient’s suffering but can also lead to increasing and possibly irreversible joint destruction. It also increases the costs to the healthcare system owing to unnecessary diagnosis, treatment, and potentially longer hospitalizations [23, 24].

Considering these reports, it is particularly important to initiate MRI diagnostics promptly in the case of persistent painful swelling and restricted movement of the elbow without traumatic event and inconspicuous infection parameters. Prakash *et al*. described specific findings on MRI that could lead to the diagnosis in 2016 [25]. If TB is suspected and synovitis is confirmed via imaging, biopsy as well as routine diagnostic testing (Table 1) should be initiated since, in approximately 40% of cases, the lung is also affected [7, 10, 26].

In about half of the cases, similar to our report, MRI diagnostics can show pronounced synovialitis accompanied by osteolysis, active and chronic pannus, and central and peripheral erosions in the area of the bone [27, 28]. However, these radiological changes can also be completely absent or occur in other diseases such as rheumatoid arthritis, therefore complicating the diagnostic process [29–32].

Bone and joint TB is mainly caused by the strain *M. tuberculosis* (> 90%). In a French retrospective analysis, *M. bovis* was also detected in 4.3% of patients, whereas a Chinese study failed to detect this strain even once [5, 27].

In about one-tenth of cases, the TB pathogen is resistant to one of the four standard drugs (isoniazid, rifampicin, pyrazinamide, and ethambutol). Multidrug resistance (resistant to at least isoniazid and rifampicin) is found in about 2% of cases, as in our case [1, 12]. In addition, literature confirms that articular TB is more frequently caused by multidrug-resistant strains [4–6]. In 2021, 77 cases of multidrug-resistant (MDR) TB were recorded in Germany [12].

With regard to therapy, it has been shown that joint TB can be treated effectively with an antibiotic therapy in accordance with the resistogram [7]. The duration of therapy should be adapted to the clinical and radiological course of events. Controls should occur every 6–12 weeks. The initial therapy should consist of a four-drug regime for 2–3 months, followed by 6–9 months of sustaining therapy. Some authors recommend an extension of this treatment for up to 18 months [33]. A Greek retrospective analysis described a median duration of therapy of 10.8 months, with a 75% success rate in bone and joint TB [26].

According to the current guidelines, a therapy duration of 9 months is recommended for bone and joint tuberculosis: fourfold therapy with isoniazid (INH), rifampicin (RMP), pyrazinamide (PZA), and ethambutol (EMB) over 2 months and then twofold therapy with INH, RMP over 7 months [11].

Thus, in our case, we observed a steady decrease of synovial changes in the follow-up MRI controls and a good clinical outcome based on the collected scores. Surgical treatment should be evaluated carefully and only be used as a last resort or when antibiotic therapy does not lead to a significant improvement [34]. This has already been described in the past by Martini *et al*. [35], in their publication from 1980, where acceptable mobility could be achieved by targeted antibiotic therapy over 12 months.

In their recent review, Upadhyaya *et al*. conclude that surgical treatment (debridement, arthrodesis, arthroplasty, or arthroscopy) should be attempted in the absence of clinical and radiological improvement after 4–6 weeks of drug therapy, in the case of infected lesions close to the elbow with a risk of joint collapse and in the case of Kerri and Martini stage III–IV, with preserved 20° range of motion [8].

In contrast, Qian *et al*. reported very good results in their retrospective analysis of six patients treated with open arthrolysis and a motion fixator [36]. All patients received the antibiotic therapy for 6–12 months after their discharge. The mean preoperative range of motion (ROM) in the study was 50.8° and the Mayo Elbow Performance Score (MEPS) was 43.3 points and could be increased to 111.7° and 92.5 points after surgical treatment. When comparing our results with the abovementioned clinical results, we found that the destruction of the joint had already significantly progressed in the patients reported by Qian *et al*. In addition, three of the patients had already developed heterotopic ossifications [36]. While Qian *et al*. found surgical treatment to be helpful, Chen *et al*. described worsening of mobility after surgical treatment in 23 of their 27 examined patients, depending on the stage [9]. In contrast to the abovementioned studies, we were able to achieve a good functional outcome with drug therapy without the need for further surgical treatment, despite delayed diagnosis and the onset of osteolysis.

Overall, the key to effective therapy is the need for early recognition of the disease to counteract irreparable damage to the joint. In addition, it is important to notice that the goal of surgical therapy is to preserve joint function rather than to treat the infection [7, 8].

It should be borne in mind that this is an individual case report with individual experiences.

**Fig. 6:**
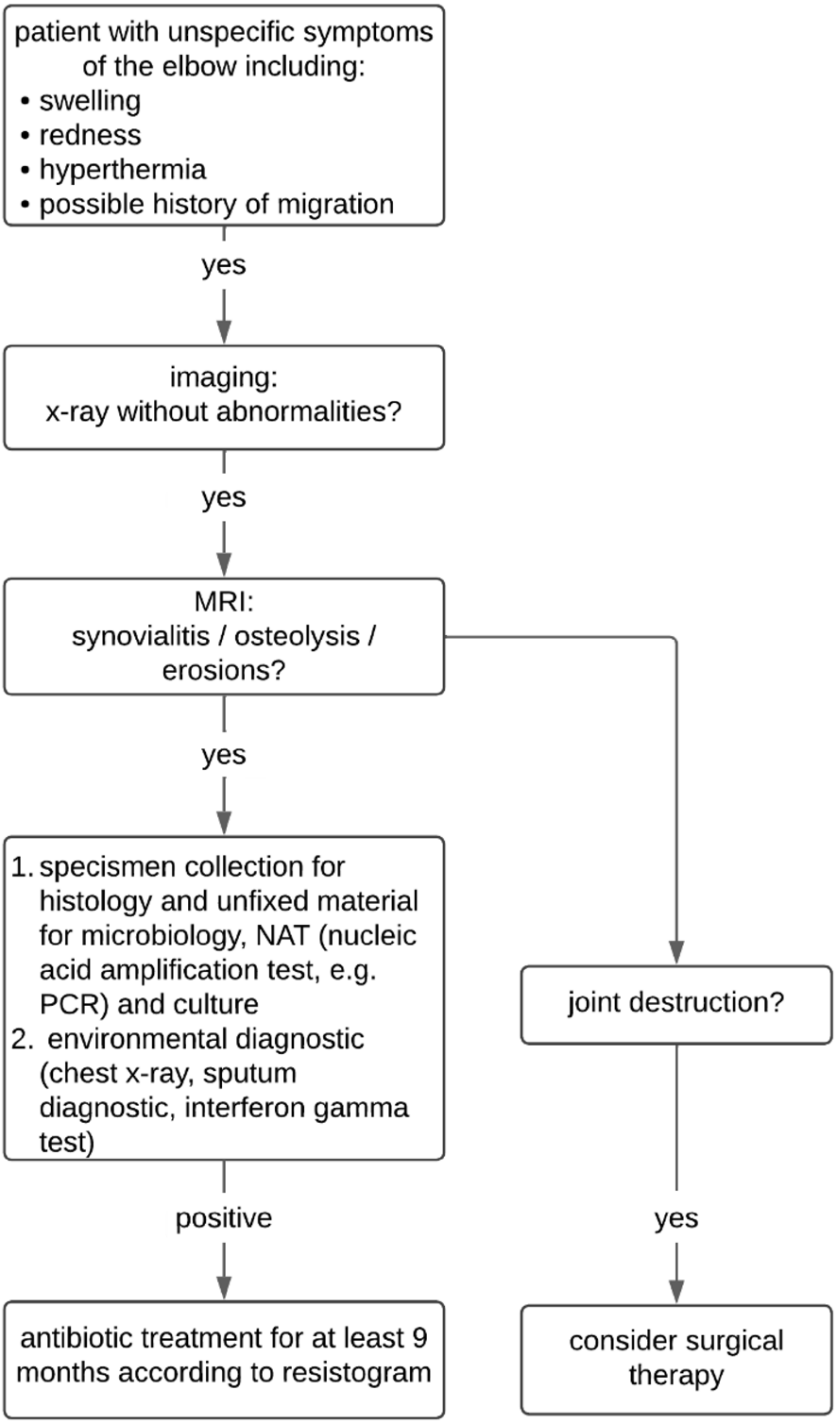
Treatment sequence for nonspecific elbow symptoms under consideration for tuberculosis

The flowchart (Fig. 6) below shows a recommended treatment sequence based on literature research.

## Conclusion

Elbow TB is a rare manifestation of TB. A differential diagnosis of articular TB should be considered at an early stage in case of unclear elbow pain. In case of early diagnosis, good clinical results can be achieved with the help of resistogram-based antibiotic therapy for about 9–12 months, depending on the level of resistance. In advanced cases with pronounced joint destruction, supplementary surgical therapy can preserve function. This, however, needs to be considered carefully.

## Data Availability

The datasets used and/or analyzed during the current study are available from the corresponding author on reasonable request.

## Abbreviations

a.p.: Anterior posterior
CrP: C-reactive protein
CT: Computed tomography
DASH: Disabilities of Arm, Shoulder and Hand Questionnaire
ECG: Electrocardiogram
ER: Emergency room
Flex/Ext: Flexion and extension according to the neutral zero method
i.e.: that is
MEPS: Mayo Elbow Performance Score
MRI: Magnetic resonance imaging
MDR: Multidrug-resistant
NSAIDS: Nonsteroidal antiinflammatory drugs
OES: Oxford Elbow Score
P/S: Pronation and supination
ROM: Range of motion
S2k guideline: Formal consensus has been reached
TB: Tuberculosis
VAS: Visual analog pain scale
